# The Management of Parkinson’s Disease: An Overview of the Current Advancements in Drug Delivery Systems

**DOI:** 10.3390/pharmaceutics15051503

**Published:** 2023-05-15

**Authors:** Deepa D. Nakmode, Candace M. Day, Yunmei Song, Sanjay Garg

**Affiliations:** Centre for Pharmaceutical Innovation, University of South Australia, North Terrace, Adelaide, SA 5000, Australia

**Keywords:** Parkinson’s diseases, nanoparticles, motor fluctuations, drug delivery, sustained release

## Abstract

Parkinson’s disease (PD) has significantly affected a large proportion of the elderly population worldwide. According to the World Health Organization, approximately 8.5 million people worldwide are living with PD. In the United States, an estimated one million people are living with PD, with approximately 60,000 new cases diagnosed every year. Conventional therapies available for Parkinson’s disease are associated with limitations such as the wearing-off effect, on-off period, episodes of motor freezing, and dyskinesia. In this review, a comprehensive overview of the latest advances in DDSs used to reduce the limitations of current therapies will be presented, and both their promising features and drawbacks will be discussed. We are also particularly interested in the technical properties, mechanism, and release patterns of incorporated drugs, as well as nanoscale delivery strategies to overcome the blood–brain barrier.

## 1. Introduction

Parkinson’s disease (PD) is the second-most-commonly occurring neurodegenerative disorder, developing between the age of 55 and 65 years [1]. The rate of disability and death due to PD is increasing faster than any other neurological disorder. In the last 25 years, PD prevalence has doubled. According to 2019 global estimates, there were approximately 8.5 million people worldwide who had PD [1]. PD is slow and prolonged, and characterized by bradykinesia, tremors, and rigidity [2]. The prevalence of PD is estimated to rise by more than 30% by 2030, significantly affecting society and the economy [3]. Presently, there is no cure for PD. Fortunately, symptomatic relief can be provided by a variety of medications such as levodopa (L-DOPA), and dopamine (DA) agonists [4]. In addition to this, research on the management of PD, targeting new methods of treatment along with continuous improvements of the existing treatment, is conducted by many research groups globally.

**Figure 1:**
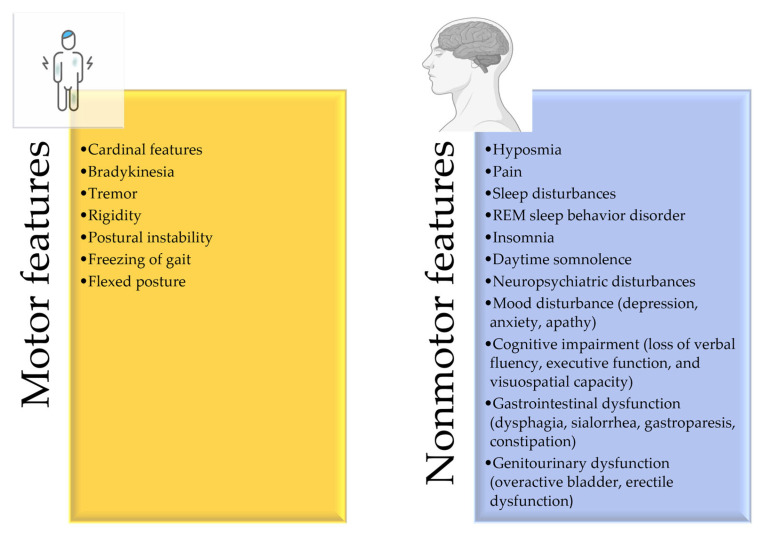
Clinical features of PD.

The occurrence of PD is more common in male populations compared to females [5]. This male predominance may be explained by the protective effects of gender-associated genetic mechanisms, the activity of female sex hormones, or gender-specific exposure to environmental risk factors [6]. The etiology of PD is complex, and several observations suggest that genetic mutations and environmental conditions might be the cause of this disease [7]. The prevalence appears to vary, either within subgroups distinguished through genotype, race, ethnicity, environment, and lifestyle related to dietary exposure to persistent organic pollutants, or by genetic characteristics that run-in families [8,9]. The clinical presentation of PD is based on the motor and non-symptoms demonstrated in Figure 1. With the progression of the disease, the severity of symptoms also increases [3].

Since PD is a very complicated disease, to efficiently conduct new curative or management research it is important to obtain a thorough knowledge of the disease mechanism and risk factors, as well as its diagnosis. Therefore, this review begins with a summary of established options for treating PD. We also discuss the most current novel nano-enabled formulations employed to obtain patient-specific therapy, with lower adverse effects compared to conventional therapies. Most importantly, to the best of our knowledge, we unprecedentedly reported for the first time in this work different marketed formulations for PD, along with their limitations and the potential solutions for overcoming those limitations. These components will be discussed in detail in the following section.

## 2. Disease Overview and Risk Factors 

### 2.1. Pathophysiology

**Figure 2:**
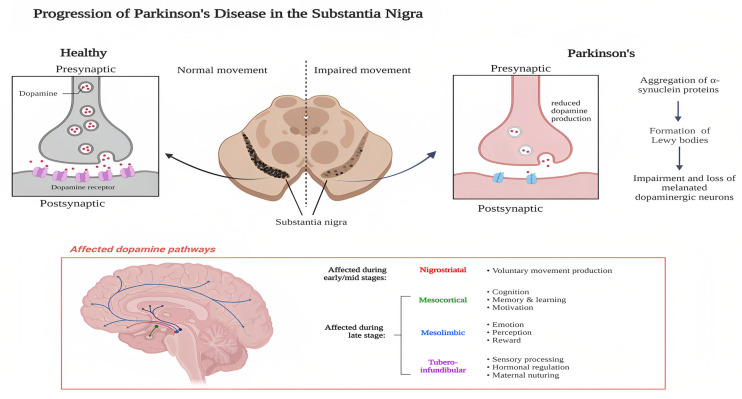
Pathophysiology of PD. Created with Biorender.com (accessed on 20 February 2023).

PD is caused by the death of DA-producing neurons in the substantia nigra, leading to a decrease in DA levels in the caudate nucleus and putamen [10]. The disease results from various mechanisms, including α-synuclein aggregation, mitochondrial dysfunction, and neuroinflammation. The Braak hypothesis explains the neuropharmacological development of PD, with the olfactory bulbs and nucleus and the dorsal motor nucleus being the earliest alterations in Braak stage 1 [11]. Braak stages 3 and 4 mark the onset of cell loss and inclusion formation in the substantia nigra, leading to clinical signs of PD in stage 3. The selective loss of DA neurons and the accumulation of Lewy bodies made up of misfolded α-synuclein are the two major pathological processes that serve as the basis for PD diagnosis, as per Figure 2 [12].

The communication between the neurons presents in the basal ganglia and the neurons of the substantia nigra (SN) occurs due to the release of DA neurotransmitters. This biochemical interaction fine-tunes organisms’ movement. The gradual degeneration of substantia nigra neurons reduces the amount of DA released for neurotransmission in the corpus striatum. As a result, PD is classified as a neurological condition that impairs movement. 

### 2.2. Etiology

Even though the exact etiology of PD is unknown, it is believed that it results from a complicated combination of hereditary variables and lifelong exposure to environmental factors such as pesticides, solvents, and air pollution [4,13]. 

### 2.3. Age 

Pathologically speaking, aging is linked to the loss of pigmented neurons in the substantia nigra, in such a manner that is distinct from PD patients [5]. PD is distinguished from aging normally by asymmetric motor symptoms and a high frequency of symptom development.

### 2.4. Pesticides and Herbicides 

MPTP(1-methyl-4-phenyl-1,2,3,6-tetrahydropyridine) was identified for the first time in 1983 for causing nigrostriatal degeneration when numerous people experienced the characteristic indications of PD after getting an injection contaminated with MPTP [14]. The discovery of MPTP as a factor in nigral degeneration gave rise to the hypothesis that environmental toxins could account for PD. 

### 2.5. Genetic Predisposition

Most of the patients who have been diagnosed with PD have no prior family history of PD, and only 10–15% of the cases reported having a family history despite the clear route of inheritance remaining unknown [15]. In addition to this, there are 23 genetic loci linked to autosomal dominant or recessive parkinsonism. However, a familial pattern can also occur through general environmental exposures [16]. PD can be challenging to diagnose accurately since patients with genetically determined PD share many clinical characteristics with those who have sporadic PD. The genetic basis of PD includes monogenic causes that are inherited either dominantly (SNCA, LRRK2, VPS35, EIF4G1) or recessively (PARK2, PINK1, DJ-1).

## 3. Clinically Approved Therapies for PD

**Figure 3:**
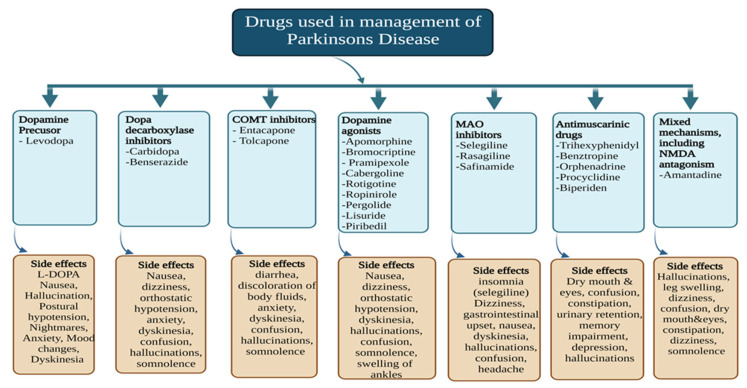
Different classes of drugs used in the management of PD.

It is difficult to deal with both the motor and non-motor symptoms of PD at both the initial and advanced phases of the disease. Therefore, the currently available treatment is not advantageous for long-term use. The primary treatment option includes medications that raise striatal DA levels or activate DA receptors, for the treatment of motor symptoms [17]. Most PD medications are now offered as transdermal patches, extended-release pills, and tablets. However, none of these formulations can deliver a drug for a prolonged period, and they require daily administration [18]. Unfortunately, none of these methods offers a long-term solution because they all become less effective as dopaminergic neurodegeneration worsens over time [19]. For advanced PD, non-invasive treatment should concentrate on optimizing dopaminergic therapy, considering parameters including absorption, timing, dosage(s), and pharmacokinetic and delivery modifications [20]. The available treatments for PD based on its mechanism, along with the side effects, are presented in Figure 3 below.

### 3.1. DA Precursor 

**Figure 4:**
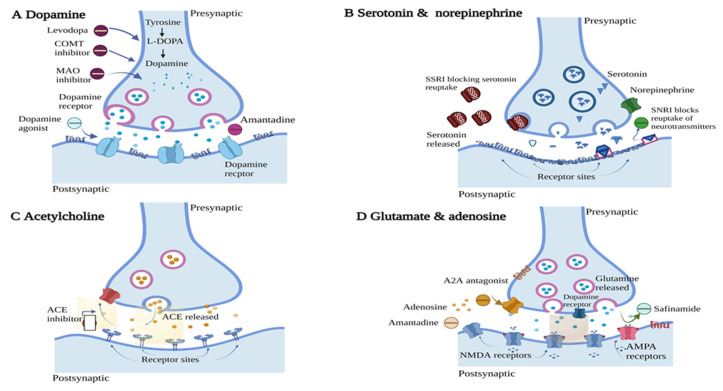
Sites of action of drugs used for the management of PD. (**A**) DA receptor—DA precursor and agonists act by stimulating DA receptor by increasing the level of DA, (**B**) Serotonin and norepinephrine—for treating depression in PD patients, serotonin reuptake inhibitors act by preventing the reuptake of serotonin at the receptor site, (**C**) Acetylcholine—Anticholinergics act as an antagonist at acetylcholine receptor, (**D**) Glutamate and adenosine—Adenosine antagonists act at this site by blocking the stimulation of adenosine receptor; this helps in treating motor symptoms. Image adapted from Bloem et al. [10] The Lancet, 2021. Created with Biorender.com (accessed on 20 February 2023).

L-DOPA is the precursor of DA, introduced first in 1967 by George Cotzias as a therapeutic agent for PD [21]. Since then, it has been established as the gold standard treatment for PD and it has become an essential component of combination therapy [22]. As illustrated in Figure 4, after crossing the blood brain barrier (BBB), the conversion of L-DOPA to DA is triggered by aromatic L-amino acid decarboxylase (AADC) through the activation of the postsynaptic dopaminergic receptor [23]. L-DOPA is prescribed by physicians to increase the level of DA in PD patients. With time, the efficacy of L-DOPA tends to decrease with dyskinesia, with “on-off” phases occurring in more than 80% of patients receiving treatment for more than 10 years [24]. The “on” period is when L-DOPA is working well and symptoms are controlled, whereas the “off” period is when medication is no longer effective and symptoms including tremors, rigidity, and slow movement return. Carbidopa (CD) is frequently administered in conjunction with L-DOPA to decrease the systemic metabolism of L-DOPA and improve its level in the brain. This allows lower doses of L-DOPA to be given to minimize side effects such as nausea [19].

**Figure 5:**
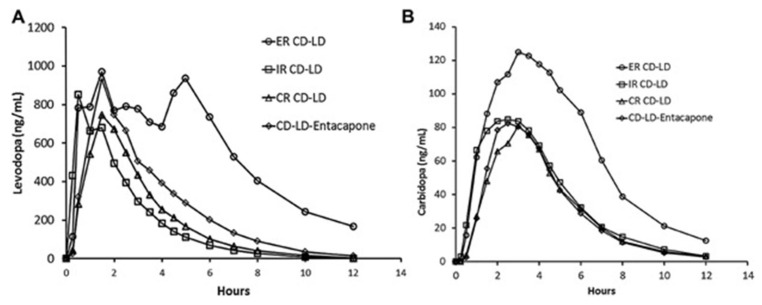
Average L-DOPA (**A**) and CD (**B**) plasma concentration of extended-release (ER) CD- L-DOPA (CD- L-DOPA) (97.5 mg CD–390 mg L-DOPA), immediate-release (IR), CD- L-DOPA (25 mg–100 mg), controlled-release (CR) CR CD- L-DOPA (25 mg–100 mg), and CD- L-DOPA -entacapone (25 mg–100 mg–200 mg) under fasting conditions. Reprint with permission from Hsu et al. [30] under Creative Common License. John Wiley and Sons, 2015.

The fundamental challenge a clinician faces when managing a patient undergoing L-DOPA therapy is preserving the symptomatic benefit of patients, or “ON” time, while preventing peak-dose dyskinesia, commonly shown as chorea, dystonia, or myoclonus, in addition to limiting wearing-off effects [25]. Whatever the motor symptoms observed due to L-DOPA therapy, they can be correlated with the duration for which the patient was on L-DOPA therapy and disease duration. Usually, PD patients with a younger onset are more susceptible to these issues than those with late-onset PD [26]. Due to the very short half-life of L-DOPA (about 90–120 min), treating motor symptoms frequently requires individual doses that inversely decrease with increasing frequency of administration. To enhance the pharmacokinetics of L-DOPA, numerous alternative formulations of the drug have been designed. For example, the effects of L-DOPA can be prolonged using the first ER (Extended Release) product, Sinemet CR (Merck Co., Morgantown, WV, USA), which was marketed in the 1980s. The components of Sinemet CR include L-DOPA, CD, hydroxypropyl cellulose, polyvinyl acetate-crotonic copolymer, magnesium stearate, and red ferric oxide, where hydroxypropyl cellulose acts as a polymeric matrix providing controlled drug release, as it erodes slowly after administration [27]. The systemic bioavailability of Sinemet CR was less compared to the immediate-release tablet, which necessitates an increased dose to achieve symptomatic relief. However, this medication has led to inconsistent absorption (4–6 h), inadequate or delayed response, and the exacerbation of peak-dose dyskinesias [28]. To address this issue, the long-acting CD- L-DOPA medication Rytary ER capsule (Impax Laboratories, Inc., Hayward, NJ, USA) was developed to alleviate dyskinesias and motor fluctuations, while attempting to retain patients’ “on” time [29]. The Rytary capsule provides immediate as well as delayed release due to the presence of immediate-release beads containing croscarmellose as a super disintegrant and delayed beads containing hypromellose as a polymeric matrix controlling the drug release. However, Hsu et al. [30] carried out a randomized, single-dose trial comparing the pharmacokinetic profile of immediate-release Sinemet and sustained-release Sinemet CR, extended-release CD- L-DOPA (Rytary) and CD- L-DOPA -entacapone (Stalevo^®^) [30]. The study found that the extended-release (ER) formulation of CD and L-DOPA (CD- L-DOPA) provided a similar initial increase in L-DOPA concentration as the immediate-release (IR) formulation but maintained the concentration at more than 50% of the maximum level for an additional 1.9 to 2.5 h compared to other CD- L-DOPA products (Figure 5).

Furthermore, intestinal gel L-DOPA is available under the brand names Duopa (AbbVie, Inc. in the USA) and Duodopa (AbbVie, Ltd. in Europe), which is a suspension of L-DOPA (20 mg/mL) and CD (5 mg/mL) in carmellose sodium administered via an tube inserted directly into the jejunum, as the absorption of L-DOPA is rapid from the intestine. Its objective is to eliminate L-DOPA peaks and troughs through continuous treatment via oral administration [31]. 

Table 1 below demonstrates all the approved conventional and novel marketed formulations of L-DOPA along with the limitations of each therapy, while Table 2 presents preclinical formulations of L-DOPA.

L-DOPA-induced motor complications are primarily induced by the pulsatile activation of striatal DA receptors [57]. The release of DA follows both tonic and phasic ways, under normal conditions. Due to the tonic firing predominance, the DA reuptake systems start working rapidly and the synaptic end experiences a consistent level of DA, leading to the continual stimulation of DA receptors [58,59]. The risk of irregular pulsatile receptor stimulation occurs due to the loss of striatal dopaminergic terminals, which affects their capacity to store DA and control its release, and the intermittent administration of a dopaminergic drug with a short half-life [60]. During the early phase of PD, enough DA terminals are present in the brain to store the DA and balance the variations in the plasma level of L-DOPA and allow the relatively constant and physiological activation of DA receptors. With the progression of the disease, the dopaminergic terminals degenerate over time, and the brain’s DA levels become reliant on the presence of peripheral L-DOPA. Consequently, the variations in the plasma concentration of L-DOPA cause fluctuations in the levels of L-DOPA and DA in the brain, exposing DA receptors to alternating concentrations of DA. The idea that L-DOPA motor problems are caused by the pulsatile activation of striatal DA receptors is supported by a significant number of studies. For instance, when supplied infrequently, L-DOPA, given as the more-stable and more-soluble prodrug L-DOPA methyl ester, causes motor-response shortening in parkinsonian rodents, but not when given continuously [61]. In MPTP-treated monkeys, L-DOPA and short-acting DA agonists have a higher probability of causing dyskinesia compared to long-acting dopaminergic drugs [62,63,64,65]. Pulsatile administration of the same short-acting experimental DA agonist causes dyskinesia, whereas continuous administration does not [66]. It has also been proposed that treatments that give more-continuous DA receptor stimulation will have anti-parkinsonian benefits and a lower likelihood of causing motor problems [67]. Preclinical studies involving how L-DOPA and L-DOPA-induced dyskinesia can be alleviated are summarized in Table 2 below.

**Table 2:** Other preclinical formulations of L-DOPA.

Formulation	Drug	Polymer	Characteristic Feature	Route of Administration	Reference
Nanoparticle	L-DOPA, curcumin	Block polymer consisting of polyethylene (Average molecular weight (M.w) 5 × 10^3^ Da) and polycaprolactone (Average M.w 10.5 × 10^3^ Da)	Enhancement in the potential of nanoparticles to cross the BBB	NA	[68]
Nanoparticle	L-DOPA	D,L-PLGA_50:50_ (M.w 7000–17,000 Da)	The intranasal formulation was prepared; extended release of up to 9 h was observed.	Intranasal	[69]
Crystalsomes	L-DOPA	L-PLGA (M.w 12,000 Da)	L-DOPA crystalsomes showed release for a longer period compared to traditional L-DOPA nanoparticles	Intravenous	[70]
Nanogel/ Hydrogel	L-DOPA	ĸ-Carrageenan	pH-dependent drug release was obtained for >11 days	NA	[71]
Carboxylated carbon nanotubes	L-DOPA	Single-walled carbon nanotube, chitosan (low molecular chitosan with 75–85% degree of deacetylation)	A surface coating helps in reducing the dosing frequency without increasing the dose of the drug	Oral	[72]
Microparticles	L-DOPA methyl ester, benserazide	D,L-PLGA _50:50_ (M.w 47,000 Da)	Microspheres showed sustained release without burst release. Reduction in dyskinesia was observed due to continuous stimulation	Subcutaneous	[73]
Microspheres	L-DOPA-α-lipoic acid	D,L-PLGA _50:50_ (intrinsic viscosity- 0.41 dL/g)	Reduced fluctuation in the level of L-DOPA in plasma	Subcutaneous	[74]

PLGA = Poly Lactic-co-Glycolic Acid.

### 3.2. Disease-Modifying Treatment 

Disease-modifying treatments for PD aim to slow down or halt the neurodegenerative process by protecting and preserving the remaining DA-producing neurons in the brain. These treatments may involve drugs that target specific biological pathways involved in the disease process, such as alpha-synuclein, which is a protein that accumulates in the brains of PD patients [75]. While there is currently no cure for PD, disease-modifying treatments have the potential to delay the onset of motor symptoms and improve the quality of life of patients with the disease. As mentioned previously in this review, the currently available treatment for PD only offers symptomatic relief, and none have been proven to reduce or prevent the disease’s progression. Therefore, the disease-modifying treatment mainly focuses on reducing α-synuclein toxicity, either by lowering α-synuclein aggregates, promoting α-synuclein clearance, or stopping the transmission of dysfunctional α-synuclein from cell to cell [76]. The sections below provide additional information about these treatment approaches. 

#### 3.2.1. Small Molecules

NPT-200-11 is a novel small entity that acts by targeting the diseased α-synuclein—the protein that is linked to PD—by stabilizing its conformation and preventing it from assembling into harmful pore-like oligomers [77]. Neuropore therapies and UCB finished a Phase I trial evaluating the pharmacokinetics, safety, and efficacy of NPT-200-11 in healthy individuals [78]. Some of the preclinical studies reported that α-synuclein aggregation can be reduced by inhibiting c-Abl a tyrosine kinase [78]. The overexpression of c-Abl enhances the aggregation of α-synuclein, whereas its inhibition reduces the aggregation of α-synuclein in vivo [79]. 

Nilotinib (Tasigna) is currently approved in the US for the treatment of imatinib-resistant chronic myelogenous leukemia (CML). Nilotinib has been demonstrated to increase amyloid clearance, providing a promising method for lowering levels of α-synuclein [80]. Nilotinib is a c-Abl inhibitor, an oncogene that controls cell development, differentiation, proliferation, and survival. PD has been associated with elevated levels of c-Abl, which is believed to cause an increase in the phosphorylation and aggregation of α-synuclein [81,82]. Additionally, a number of the treated individuals reported improvements in their clinical symptoms; nevertheless, it is crucial to remember that this was an open-label trial with no placebo group [83]. 

At present, regulating the activity of the glucocerebrosidase (GBA) pathway is another mechanism being investigated for the degradation of α-synuclein. The main genetic risk factor for sporadic PD is mutations in GBA, and it is believed that decreased GBA activity causes an accumulation of α-synuclein [84]. Ambroxol is an anti-mucolytic medication that has recently been demonstrated to increase GBA levels and the activity of fibroblasts in both controls (without GBA mutant carriers) and GBA mutant carriers [85]. 

#### 3.2.2. Repurposing of Other Drugs

In toxin-based mouse models of nigrostriatal degeneration, exenatide-a glucagon-like peptide 1 agonist has been demonstrated to have neuroprotective and neurorestorative effects, providing improvements in motor function, behavior, learning, and memory [86,87]. In patients with moderate PD, the effects of subcutaneous exenatide were investigated in a recent double-blinded placebo-controlled experiment [88]. After 60 weeks, the treatment was attributed to both beneficial and long-lasting improvements in off-medication motor scores. Although the follow-up period in the PD context is brief, and it Is still unknown if this treatment slows the progression of neurodegeneration in PD, though these preliminary findings provided promising potential for the treatment of PD.

#### 3.2.3. Immunotherapies

Extracellular α-synuclein has been targeted and degraded using active and passive immunotherapies, which were demonstrated to diminish α-synuclein aggregation and prevent behavioral abnormalities in transgenic mice [89]. Several clinical trials have begun, and early safety data and outcomes are encouraging [90]. For example, the active immunotherapeutic (AFFITOPE PD03A), developed by AFFiRis AG, has completed the Phase 1 trial [91]. This vaccine was created synthetically and contains a peptide that mimics alpha-synuclein. In this study, low and high concentrations of AFFITOPE PD03A were repeatedly injected subcutaneously into patients with early-stage PD. Positively, both doses were well-tolerated, and there were no major adverse medication reactions observed. Furthermore, AFFITOPE PD03A has been found to be safe in the initial stages of PD for over 4 years. Additionally, passive immunotherapy using a humanized anti-α-synuclein antibody (PRX002) has also been investigated. For example, a Phase Ia clinical trial by Prothena showed a decrease in the levels of α-synuclein up to 96.5% in healthy volunteers [92]. Subsequently, in PD patients, a Phase 1b trial showed a reduction in α-synuclein levels [92]. Most importantly, a recently conducted Phase II investigation in patients with early-stage PD has concluded that PRX002 is safe and well-tolerated [93]. 

#### 3.2.4. Gene Transfer/Viral Vector-Mediated

Utilizing viral vectors to achieve the expression of genes in the striatum is another promising strategy for a regenerative treatment for PD. These strategies make use of the lentivector expression system and the adeno-associated viral vector serotype 2 (AAV2) for the transfer of the foreign gene into the host genome [94]. Trials involve several approaches for preventing neurodegeneration and related clinical outcomes through (a) the delivery of the L-DOPA-converting enzyme L-amino acid decarboxylase (AAV2-AADC); (b) delivering neurotransmitter gamma-aminobutyric acid-producing enzymes (AAV2-GAD-65, AAV2-GAD-67); (c) the delivery of the tricistronic lentivector to transfer three genes, including tyrosine hydroxylase (TH), GTP cyclohydrolase-1 (involved in DA synthesis), and AADC; and (d) using genes that provide trophic development in the putamen and substantia nigra, such as neuturin (AAV2-NRTN) and glial cell-derived neurotrophic factor (AAV2-GDNF) [95,96,97]. PD-1101 was a Phase 1b trial that evaluated the safety and efficacy of VY-AADC01 delivered bilaterally to the putamen in PD patients. VY-AADC01 is an adeno-associated virus serotype 2 (AAV2) gene therapy that encodes the human AADC (hAADC) enzyme, which is designed to increase DA production by delivering the AADC gene directly to the putamen [98]. The results of the PD-1101 trial, which evaluated the safety and efficacy of VY-AADC01 gene therapy delivered to the putamen in patients with moderately advanced PD and motor fluctuations, demonstrate that the therapy and surgical delivery procedure were well-tolerated, with no serious adverse events related to the vector reported over a three-year follow-up period. Furthermore, the safety and tolerability of VY-AADC01 were observed even with infusion volumes that were several times higher than those used in previous AADC gene therapy trials in PD. More research needs to be conducted to for prove the effectiveness of motor functions [98]. Another open-level Phase 1 study, evaluating the safety, tolerability, and efficacy of the intraputaminal delivery of CERE-120 (AAV2-neurturin), was performed in PD patients. The delivery of CERE-120 was found to be safe and well-tolerated, with no serious adverse effects reported in the small number of patients studied. In the Phase 2 trial, serious adverse effects were observed, which were mainly due to surgery. Such a delivery system offers limited cargo size and requires surgery for administration, which is not feasible for all patients [99].

## 4. The Special Emphasis of Nanoformulations on Controlled Drug Delivery Systems for PD Management 

With decades of clinical use of already available medications in PD, a variety of therapies have been introduced. However, the delivery of the drug to the central nervous system (CNS) remains a huge obstacle in providing effective treatment for PD patients, due to the protective mechanism of the BBB. Growing interest has been shown in the design of micro- and nanosystems that can improve drug transport to the brain through their ability to bypass the BBB, enhancing the pharmacological and therapeutic characteristics of both conventional and novel drug molecules. Micro-/nano-drug delivery systems (DDSs) can be used for localized treatment through direct administration to the brain, or systemic delivery for targeted delivery to the CNS. Depending on the type of material and the substance used to prepare it, different DDSs may be either biodegradable or non-biodegradable. Several preclinical studies have reported the different micro-/nano-DDSs utilized to deliver drugs or small molecules to the brain, with varying degrees of success in providing neuroprotection (Table 3). The modification of drugs at the nanometric scale might bring about an improvement in the half-life and bioavailability of therapeutic compounds, attaining a sustained drug-release drug profile [100]. 

In addition to the parenteral route of administration, buccal or subcutaneous routes are considered promising alternatives, as the time of onset and bioavailability of dopaminergic drugs delivered via these routes can be greatly improved [101]. Intranasal administration is another popular route used to deliver micro-/nano-enabled drugs to the CNS, through olfactory and trigeminal pathways [102]. The intranasal route facilitates bioavailability improvement and dose reduction of therapeutic molecules, owing to its ability to bypass the BBB [103]. In addition to this, since many PD patients experience dysphagia as their condition worsens, significant work has been put into developing novel formulations of these medicines to treat these conditions, allowing their delivery at lower concentrations (Table 2) (e.g., inhaled L-DOPA). In the section below, preclinical studies carried out using different formulation approaches to overcome the limitations of existing treatments, together with their claimed positive outcomes, will be discussed in detail.

### 4.1. Polymeric Nanoparticles

**Figure 6:**
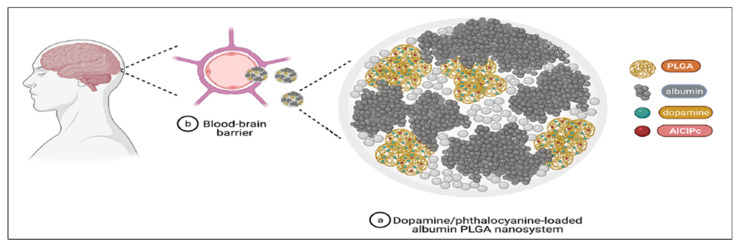
Diagrammatic depiction of DA/phthalocyanine-loaded nanoparticles (**a**) as they cross the BBB (**b**) to ultimately enter the brain parenchyma and liberate the incorporated drug molecules. Albumin nanoparticles possess the ability to cross the BBB by receptor-mediated transport 147 [111]. AICIPc—aluminum chloride phthalocyanine. A fluorescence marker was used for tracking the movement of the nanosystem to cross the BBB after administration 148 [112]. The prepared DA nanosystem could cross the BBB, which was tracked by fluorescent marker AICIPc. Reprint with permission from Monge-Fuentes et al. under a Creative Commons license [110]. Nature, 2021.

Amongst the wide range of nanomaterials used in theranostic drug delivery, nanoparticle-based technology is considered a promising choice for treating disease [104,105]. Specifically, by surface modification, polymeric nanoparticle facilitates drug transport to the brain by targeting or crossing the BBB through receptor-mediated transport. This strategy allows the delivery of BBB-impermeable drugs such as DA without affecting the brain integrity and its normal function, thus attracting special attention [106]. Since the hydrophilic nature of DA hinders the delivery of DA through the BBB [107], various nanoformulation approaches have been explored to enhance the transportation of the drug across the BBB, increasing its bioavailability, efficacy, and stability. For example, several attempts have been made by incorporating DA into D,L-PLGA-included nanoparticles, with positive outcomes such as reducing anxiety, enhancing coordination, and increasing physical movement in animal models [106,108,109]. This is evident in the recently published study by Fuentes et al., who encapsulated DA in albumin/D,L-PLGA (ALNP) nanoparticles for improving DA transport to the brain. The fluorophore marker (aluminum chloride phthalocyanine) was included in this ALNP-DA system for the tracking of ALNP transport in the brain (Figure 6). The neurotherapeutic potential of DA-loaded ALNP was assessed in the 6-OHDA-induced PD model in mice (lesioned group). In comparison to the lesioned and native L-DOPA groups, ALNP-DA successfully crossed the BBB, replenishing DA at the nigrostriatal pathway and leading to a substantial improvement in motor symptoms. Remarkably, when ALNP-DA was dosed at 20 mg/animal, it additionally upregulated and restored balance, sensorimotor function, and motor coordination to the same levels as observed in non-lesioned (Sham) animals. Considering ALNPs’ ability to penetrate the BBB and finally transport the intended drug to the brain, they constitute a novel, non-invasive, nano-therapeutic approach for treating PD [110].

In addition to this, age-related neurodegenerative diseases are also associated with the overexpression of lactoferrin receptors in the capillaries and neurons. Therefore, Bi et al. prepared an intranasal formulation of rotogotine-loaded PEG-D,L-PLGA nanoparticles modified with lactoferrin (Lf-NPs), which is an iron-binding glycoprotein. Prepared formulations showed sustained drug release for up to 48 h in a 7.4 pH buffer. The results of qualitative and quantitative cellular uptake studies in 16HBE and SH-SY5Y cells showed a higher accumulation of surface-modified nanoparticles than that of plain NPs. After intranasal treatment in rats with Lf-NPs, increased rotigotine concentrations in the striatum—the main area affected by PD—were observed. This suggested that Lf-NPs could deliver rotigotine from the nose to the brain for treating PD [113]. 

The application of biodegradable polymers such as chitosan in the preparation of solutions, hydrogels, and nanoparticles has drawn special interest in intranasal drug delivery. Sridhar et al. [114] prepared a novel intranasal chitosan nanoparticle (NPs) system loaded with selegiline by ionic gelation. Drug loading up to 90% was obtained, and the prepared formulation was also tested against a marketed oral tablet. The brain and plasma concentrations of selegiline given through this system were 20 and 12 times greater than that of orally administered marketed formulations. Additionally, using intranasal nanoparticles will lead to considerably higher levels of DA, catalase activity, and glutathione in the brain, as well as the improved performance of locomotor activity, catalepsy, and stride length tests [114]. Thus, selegiline nanoparticles given by the intranasal route have a higher therapeutic potential than those given orally and may represent a useful strategy for PD treatment. A similar study was reported by Sumit et al. in which a thermos-reversible intranasal gel consisting of L-DOPA -encapsulated nanoparticles was developed for enhancing brain uptake and reducing peripheral degradation [115]. Polycation possesses a mucoadhesive property that helps improve drug absorption through the nasal mucosa, both by opening a tight junction between epithelial cells and by slowing mucociliary clearance. L-DOPA -loaded chitosan NPs were prepared by an ionic gelation technique, which were incorporated into the thermos-reversible gel of Pluronic PF 127. After intranasal administration in rats, over 90% of L-DOPA concentration was recovered in the brain. The results of this study suggested that the thermo-reversible Pluronic F127 gel system as a carrier would facilitate easy intranasal delivery and prolong the formulation’s residence time.

In another study, conducted by Bali and colleagues, a D, L-PLGA NP-based transdermal patch was investigated for continuous drug delivery to the brain [116]. D, L-PLGA NPs loaded with rasagiline mesylate were prepared using the double-emulsion technique; they were embedded in gellan gum film using the solvent casting method. The optimized NPs showed a slow initial release followed by a continuous release for more than 72 h in ex-vivo diffusion studies. An in-vivo comparison of the transdermal film with oral and iv formulation showed a 7-fold and 11-fold increase in area under the curve (AUC). Hence, the results implicate an increase in the bioavailability of rasagiline by transdermal application. Gamma imaging technology was used to monitor brain targeting, which revealed an increase in drug transport to the brain through the transdermal film. This finding was also supported by increased brain targeting efficiency, determined by a biodistribution experiment [116]. All the polymeric nanoformulations intended for the treatment of PD put forward since 2015, together with their advantages, are summarized in Table 3 below.

**Table 3:** Recently reported nanoparticles studies since 2015.

Formulation Type	Drug/API	Polymer	Advantages	Route of Administration	References
Nanoparticle	Rotigotine	Maleimide-PEG -D,L-PLGA (M.w 3400–20,000 Da) and methoxy-PEG-D,L-PLGA (M.w 2000–20,000 Da)	A higher concentration of rotigotine was observed in the striatum with release up to 48 h	Intranasal	[113]
Nanoparticle imprinted film	Selegiline	D,L-PLGA, Ethylene-vinyl acetate	In-vivo drug release for more than 72 h was observed from the film	Transdermal	[117]
Nanoparticle	L-DOPA methyl ester/benserazide	D,L-PLGA_50:50_ (M.w 47,000 Da), PLA (M.w 83,000 Da)	The prepared formulation reduced the L-DOPA-induced dyskinesia in the dyskinetic rats	Subcutaneous	[118]
Nanoparticle	Pramipexole 2HCL	Chitosan (>90% deacetylation)	In-vivo and ex-vivo diffusion showed complete drug release in 24 h. Reduced motor deficit in nanoparticle-treated group compared to nasal solution and oral tablet	Intranasal	[119]
Nanospheres	VEGF	D,L-PLGA	VEGF encapsulated in the nanosphere could cross the BBB and showed a strong neuroprotective effect	Intravenous	[120]
Nanoparticle (Device)	DA	Cellulose acetate phthalate (M.w 49,000 Da)	DA was released for 30 days with a higher concentration in CSF than in plasma, which reduced the side effects of DA	Intracranial	[121]
Polysorbate 80 coated nanoparticle	Ropinirole HCL	Chitosan (M.w 150,000 Da)	In-vitro drug release up to 10 h was observed. In-vivo studies revealed the higher concentration of ropinirole in the brain over other highly perfused organs	Intravenous	[122]

### 4.2. Solid Lipid Nanoparticles (SLNs)

SLNs possess various advantages such as biocompatibility, the potential for a high loading of hydrophobic drugs, better stability compared to liposomes, and a controlled drug-release profile [123]. In addition to this, SLNs have a great potential for drug targeting by surface modifications, allowing for easy transport across the biological membranes due to their lipidic nature and small size [124,125]. Various modification approaches for SLNs (solid lipid nanoparticles) and NLCs (nanostructured lipid carriers) are utilized for promoting drug transport to the brain, including surface binding with a molecule that binds to brain receptors, hydrophilic coating, and size manipulation, which enable the circumvention of rapid clearance by the reticuloendothelial system (RES). Hence, lipid nanoparticles enable the lowering of the toxicity of incorporated drugs due to reduced contact with non-target tissues, achieving therapeutic concentration with reduced doses of therapeutic molecules. Though the particle size of SLNs and NLCs used for CNS diseases [126] ranges from 50 to 1000 nm, for the intravenous route and nose-to-brain delivery a particle size of 200 nm diameter is necessary for easy access to the CNS [127]. Unfortunately, the limitations of lipid nanoparticles include low encapsulation efficiency for hydrophilic drugs and long-term storage instability, especially in the case of thermolabile drugs [124]. Drugs such as apomorphine are linked to a lipid-based nanomolecule that has been modified to encapsulate or bind the drug inside. It improves systemic lymphatic absorption when given orally and increases the production of chylomicrons [100]. 

SLN delivery by oral route has also been explored for the delivery of Apomorphine, a DA receptor agonist. Apomorphine has a very poor bioavailability of <2%, which necessitates multiple administrations. To overcome these problems, Tsai and colleagues prepared an SLNs system using glyceryl monostearate (GMS) and polyethylene glycol monostearate (PMS) [128]. As a result, the change in emulsifiers had a significant impact on the physicochemical properties of SLNs. For example, the diameter of SLNs prepared using GMS was 155 nm, whereas the diameter of SLNs prepared using PMS was found to be 63 nm. Both SLNs successfully encapsulated more than 90% of apomorphine. In-vivo studies revealed a 12- to 13-fold increase in the bioavailability of the SLNs compared to that of the apomorphine solution. The results of the in-vivo drug distribution showed the potential of SLNs in the successful transfer of apomorphine to the brain striatum, suggesting their potential to significantly enhance the efficacy of apomorphine against PD. Interestingly, PMS SLNs were more effective than the GMS SLNs in a PD rat model in terms of a behavioral index.

For delaying the mucociliary clearance after the intranasal administration of SLNs, Uppuluri et al. prepared piribedil-loaded SLNs using palmitic as a solid lipid core and polyvinyl alcohol as a stabilizer [129]. The prepared SLNs were further incorporated into thermos-responsive methylcellulose gel for improving the intranasal delivery of piribedil. Using a cannula and microtip arrangement, intranasal delivery at the olfactory area was performed. An in-vivo pharmacokinetics study was carried out in rats, with a 4-fold higher brain uptake of piribedil obtained compared to that of the native piribedil suspension. These findings demonstrate that the developed piribedil SLNs are notably more efficacious for PD management compared to the standard oral pharmaceutical product [129].

In an experiment, Dudhipala et al. [130] prepared ropinirole-loaded SLNPs and lipid carriers for improving oral and topical delivery (for topical delivery, SLNs and NLCs were loaded in Carbopol 934 gel). SLNs and NLCs showed sustained drug release for up to 24 h in in-vitro and ex-vivo diffusion studies. The results of the pharmacokinetics study showed a 2.1-fold and 2.7-fold increase after the SLN and NLC formulation after oral administration, whereas 3- and 3.3-fold increases were observed after topical administration, compared to the native drug suspension [130]. 

Pardeshi et al., explored the surface modification of NLCs to improve the mucoadhesive property of the NLCs [131] by developing tristearin NLCs containing a ropinirole-dextran sulfate nanoplex that was previously surface-modified with N,N,N-trimethyl chitosan to extend the residence duration of the drug on the nasal mucosa. The sustained release of the incorporated drug for up to 36 h was observed in in-vivo diffusion studies. Modified NLCs demonstrated a 13.3-fold higher bio-adhesive strength compared to NLCs. A study of brain and plasma pharmacokinetics in mice models also demonstrated the potential of NLCs to carry drugs from the nose to the brain through the olfactory route. Following intranasal delivery, the Cmax and AUC (0–∞) values of modified NLCs increased by 17 and 6.5 fold, respectively, compared to ropinirole suspension, demonstrating the formulation’s improved CNS bioavailability due to the small particle size and lipophilic nature of modified NLCs [131]. 

Additionally, PEG-modified carriers showed greater stability when used as nanoparticles. This could be attributed to the ability of PEGylation to increase the time that a drug resides in the bloodstream. PEGylated nanoparticles exhibit longer half-lives and slower plasma clearance rates as compared to their conventional counterparts [132]. The findings of these study suggested that SLNs prepared using PMS are a novel strategy for the delivery of apomorphine for oral administration, providing an alternative approach to the subcutaneous route.

### 4.3. Microsphere and Microcapsules

Most of the drug formulations on the market are preferably administered orally due to their non-invasiveness and ease of self-administration [133]. Oral administration limits the absorption of some drugs due to extensive first-pass effects and rapid elimination from the body [134]. However, in the case of conventional parenteral products, after dosing, a drug enters blood circulation rapidly and then rapidly declines following the peak concentration [135]. This causes fluctuations in plasma drug concentration and requires repeated injections. In contrast to conventional parenteral dosing, the controlled-release parenteral formulations maintain the drug concentration within the therapeutic window for a longer period [136]. As a result, controlled-release formulations improve drug efficiency while simultaneously lowering the risk of severe side effects.

Microspheres are an example of an FDA-approved control release system successfully transferred from the bench scale to commercial products. Microspheres were used for the first time in the 1960s. In comparison to other controlled drug delivery systems, microspheres have a few distinct advantages, such as: (i) The ability to control the rate and duration of drug release by changing the materials and fabrication processes [137]; (ii) Better stability compared to other controlled-release systems; and (iii) Patient compliance is improved as patients require a reduced frequency of dosing.

Microencapsulation is defined as the engineering of a particle in the size range from 1 to 1000 nm in which a solid or liquid drug is encapsulated, forming a polymer shell called a microcapsule, or is dispersed in a polymeric matrix known as a microsphere [138]. A variety of polymers available for microencapsulation are obtained from natural sources, including chitosan, alginate, and collagen, or are synthesized, such as D,L-PLGA-copolymer made up of lactic and glycolic acid, PCL-polycaprolactone and PLA-polylactic [139]. These polymers help prevent the degradation of drugs and reduce the toxicity of the surrounding tissues, allowing for the manipulation of drug release for a longer time, from months to days [140,141]. Amongst the available polymers, D,L-PLGA has been known to be successfully used for the formulation of parenteral microspheres due to its biocompatibility and biodegradability [142]. A formulation strategy employed in the preparation of microspheres include: spray drying, where a solution or suspension of a drug is prepared organically and is further atomized through a nozzle, leading to the formation of microspheres due to evaporation by heat; solvent evaporation, or extraction, where the drug is dispersed in a polymer solution that is further added to a continuous phase containing an emulsifier and stirred, as the organic phase evaporates, leading to the hardening of particles; and a phase-separation or coacervation technique, where a polymer solution is separated into two immiscible liquid phases, leading to dense coacervate-phase concentrate formation in a polymer-encapsulating drug [143]. The most-used technique for microencapsulation includes solvent evaporation or extraction [144,145]. Depending on the nature of the drug and its solubility, various approaches are employed for the preparation of microspheres by solvent evaporation, such as oil-in-water (O/W) emulsion for lipophilic drugs and water-in-oil-in-water (W/O/W) double emulsification for hydrophilic drugs [146,147,148].

Microencapsulation techniques have been successfully used to develop long-acting injection products; however, like any other nanoformulations, they still have several limitations, including low drug loading, difficulty in controlling particle morphology and size distribution, and the unpredictable stability of biotherapeutics, specifically proteins, peptides and nucleic acids [149,150]. The size range of microparticles intended to be injected is from 10 to 250 µm, whereas the ideal size of 125 µm is recommended for allowing the injectability of particles, as at this size the macrophage phagocytosis and inflammation of local tissue are minimized [138,151,152]. The mechanism of drug release from microspheres possibly occurs by following pathways [153]: initially, the drug on the surface is released; secondly, drug particles from the pores of microspheres; thirdly, the diffusion of the drug through a polymeric matrix occurs, followed by diffusion through a swollen polymer and finally through polymer erosion and degradation. Together, this mechanism helps to achieve controlled drug release [154,155]. In addition to this, the control of drug release can be manipulated using different grades of polymers., but burst release is difficult to control as it also depends on the solubility of the molecule.

For the delivery of drugs across the BBB, various strategies are being developed. For example, the implantation of microspheres directly into the brain will reduce the systemic toxicity of incorporated drugs and dictate the therapeutic concentration of drugs in the specific region [156]. This same approach was applied for the delivery of vascular endothelial growth factor (VEGF) and glial cell line-derived neurotrophic factor (GDNF) in a study conducted by Herran et al. [157]. Encapsulated VEGF and GDNF were co-encapsulated in D,L-PLGA microspheres using the W/O/W double-emulsion technique. The prepared microspheres showed in-vitro controlled release for up to 31 days. However, the number of surviving TH + cells was used to measure the regeneration effects of these growth factors using a rotation behavior test. Rotation behavior tests in tested rats demonstrated an improvement in both treatment groups (treated with VEGF and GDNF). In HUVECs (human umbilical vein endothelial cells) and pheochromocytoma (PC12) cells, respectively, the biological activities of encapsulated VEGF and GDNF were examined. Additionally, compared to the control group, both groups that were treated with VEGF and GDNF microspheres displayed increased amounts of neuroregeneration/neuroreparation in the substantia nigra [157].

All microsphere-based formulations intended for the treatment of PD put forward since 2015, together with their claimed advantages, are summarized in Table 4 below.

The effectiveness of microspheres in intraperitoneal administration has also been explored by several research groups. For example, Marco and colleagues prepared rasagiline mesylate-loaded D,L-PLGA _50:50_ microspheres by o/w emulsion solvent evaporation [164]. A rodent model (rotenone-induced PD) was used to test the neuroprotective potential of rasagiline mesylate microspheres after systemic administration via the intraperitoneal route. The in-vitro drug-release profile followed zero-order kinetics (where a drug is released at a constant rate from the system) and released for up to two weeks in phosphate buffer 7.4. When compared to animals treated with rotenone, the results of catalepsy, akinesia, and swim tests in animals receiving rasagiline microspheres either in solution or within microspheres showed a reversal in descent latency. Brain slices stained with 0.5% cresyl violet revealed a specific degeneration of dopaminergic neurons in the substantia nigra (SNc) [164].

**Figure 7:**
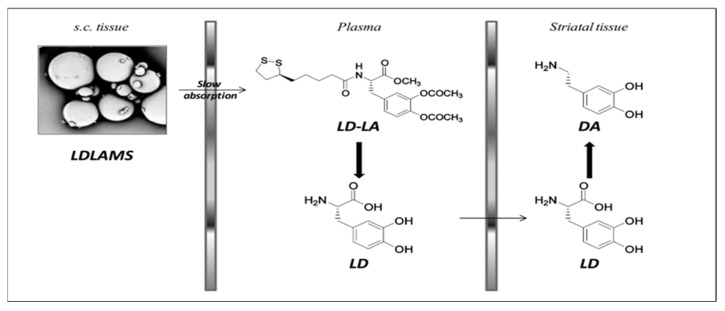
Bioconversion pathways from subcutaneous LDLAMS injection. After subcutaneous administration of L-DOPA -α-lipoic acid microspheres, prodrug LD-LA is slowly absorbed into blood circulation and is converted to L-DOPA in plasma, resulting in increased levels of DA in striatal tissue due to the bioconversion of L-DOPA to DA. Reprint with permission from D’Aurizio et al. under a Creative Commons license [74] ACS publications, 2011.

Parkinsonian patients on long-term L-DOPA therapy frequently have uncontrolled motor problems as a result of LD plasmatic level fluctuations, causing pulsatile dopaminergic stimulation [165]. L-DOPA-lipoic acid (LD-LA), a novel prodrug of L-DOPA, has been suggested by D’Aurizio et al. as a method for attaining the continuous stimulation of dopaminergic neurons to avoid plasma level fluctuation, as due to the lipophilic nature of LA it prolongs the plasma levels of L-DOPA. Nevertheless, the catechol esters and amide bonds present in L-DOPA-lipoic acid are both prone to quick chemical and enzymatic hydrolysis. As a result, repeated dosages are necessary daily to maintain effective L-DOPA levels in the brain. To address this issue and enable the prolonged release of LD-LA while also protecting this prodrug from enzymatic and chemical breakdown, this prodrug was encapsulated in the D,L-PLGA _50:50_ microsphere [166]. In-vitro prodrug release up to 1 week was observed, following Fickian diffusion, which was carried out in pH 4.5 acetate buffer. Further studies were carried out for evaluating pharmacokinetics after the subcutaneous injection of L-DOPA -α-lipoic acid microspheres; the plasma concentration of L-DOPA of 0.40 µg/mL remained constant up to 120 h. It is worth noting that after a single injection, LD-LA microspheres maintained DA neurotransmitter levels in the striatum nucleus for 4 days. As shown in Figure 7, the LD-LA microsphere after subcutaneous administration slowly releases LD-LA, which in plasma is converted to L-DOPA and further crosses the BBB. The management of symptoms using a polymeric microsphere formulation of LD-LA is appealing as it prevents motor issues without the need for comedication with benserazide [74]. 

### 4.4. Liposomes

**Figure 8:**
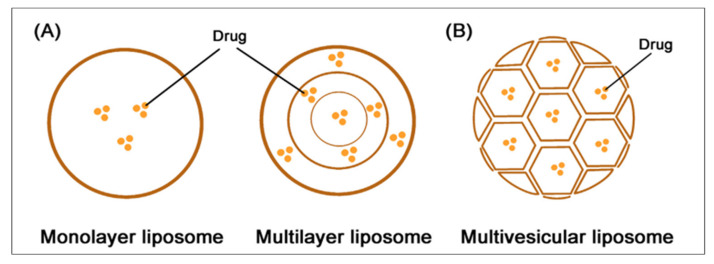
Types of liposomes. (**A**) The structure of monolayer and multilayer liposomes, where drug molecules are entrapped in the lipid layer. (**B**) The structure of multi-vesicular liposomes, with each lipid vesicle conjoined to form the pomegranate-like structure. Reprint with permission from Shi et al. [168]. Elsevier, 2021.

Liposomal delivery provides the well-established advantage of loading capacity for both hydrophilic and lipophilic cargoes, due to its lipid bilayer structure. Hydrophilic molecules are entrapped in an aqueous core, whereas lipophilic drugs are entrapped in a lipid bilayer [167]. The rate of drug release from liposomes is dependent on different packing patterns. Commonly, the packing mode of liposomes is concentric, and lipid membranes are assembled for forming a monolayer or multilayer structure [168]. The manipulation of drug release from liposomes is possible by the arrangement of lipid bilayers either into concentric circles (multi-lamellar vesicles) or non-concentric conjoined vesicles (multi-vesicular liposomes), as shown in Figure 8. The internal structure of multivesicular liposome resembles the internal structure of pomegranates, where the hydrophilic drugs entrapped in lipid vesicles are linked in the honeycomb-like structure [142]. Several studies have been carried out for evaluating liposomes as sustained drug-release systems through intramuscular and subcutaneous routes, in which efficiency was evaluated on different animal models. 

The intranasal delivery of GDNF (glial cell line-derived neurotrophic factor) provides an advantage of direct delivery to the brain by overcoming the limitations of other treatments that require surgery for their insertion into the brain. For example, nasal delivery is non-invasive, easily administrable, and retrievable, and is preferred for patients who do not qualify for surgery [169]. For example, intranasal delivery of liposome encapsulating neuroprotective GDNF, developed by Migliore et al., demonstrated potential as a non-invasive treatment for PDs. The cationic liposomes were prepared using dioleoylphosphatidylcholine (DOPC), cholesterol, and stearyl amine. The particle size of prepared liposomes was approximately 149 ± 11 nm with an encapsulation efficiency of 95 ± 1%. A significant increase in the number of tyrosine-hydroxylase (TH)-positive neurons was observed in the GDNF liposome treatment group compared to that of the control, as tested on 6-OHDA-induced rat models. DAS cell numbers were also higher in the substantia nigra of the PD model following treatment with these liposomal systems. Significant improvements in the neuroprotective efficacy of GDNF were also observed by TH immunostaining density when 150 μg doses of liposomal GDNF were given [170].

In another study reported by Xiang et al., encapsulating L-DOPA in chlorotoxin-modified stealth liposomes was carried out for targeted drug delivery [171]. In this work, liposomes were prepared by a thin-film hydration technique. The modification of the surface of liposomes demonstrated an enhanced uptake in in-vitro in murine BMECs (brain microvascular endothelial cells). The in-vivo study was performed in mice by administering the formulation intraperitoneally. The levels of DA and dihydroxyphenyl acetic acid (metabolic product of L-DOPA) were significantly enhanced in the substantia nigra and striata by L-DOPA -loaded modified liposomes, compared to that of plain L-DOPA and unmodified L-DOPA liposomes in MPTP mice models. Increased uptake by in-vitro studies could be correlated to the increased levels of DA in in-vivo studies. A good correlation was observed between in-vitro and in-vivo results, suggesting the potential of chlorotoxin-modified stealth liposomes for active targeted drug delivery for PD treatment [171]. 

In addition to this, to improve the compliance and pharmacokinetics of therapeutic molecules that are usually given orally, transdermal delivery of liposomes has also been studied as an alternative route of administration. Transdermal application of pramipexole helps in improving absorption, and patient compliance and reduces the side effects of the conventional formulation [172]. For instance, Trivedi and co-workers formulated a cationic liposome for transdermal delivery of pramipexole, indenting to reduce side effects associated with conventional pramipexole. In this work, liposomes were made charge-flexible using sodium cholate (edge activator), sodium hexadecyl sulfate (anionic surfactant), or cetylpyridinium chloride (cationic surfactant). As-prepared formulations were then incorporated into Carbopol-934 gel. A drug-release profile of up to 12 h was observed in ex-vivo studies for all the formulations. The cationic flexible liposome-containing gel showed a higher AUC with a Cmax of 163.15 ng/mL than the control gel patch, which showed a Cmax of 53.43 ± 1.97 ng/mL. As the stratum corneum is populated with anionic lipids, the use of cationic liposomes might help improve drug penetration through the skin due to electrostatic interaction. In addition to this, the interaction of liposomes with skin is regulated by the lipid content of liposomes. If fast-flowing blood levels are required, short-chain mono-substituted phospholipids can be used; whereas if drug release for longer periods is needed, then a long carbon chain can be used. 

As mentioned earlier in this review, the transport of hydrophilic APIs across the BBB is challenging when it comes to the management of PD, specifically for the transportation of DA. It does not cross the BBB due to its high polarity and ionization at physiological pH [173,174,175]. To overcome this problem, Lopalco et al. encapsulated DA in Tf (transferrin (β-1 glycopeptide)-functionalized liposomes [176]. Native liposomes were prepared by a dehydration–rehydration technique using hydrogenated soy phosphatidylcholine and cholesterol. In-vitro drug release at acidic conditions showed 59 ± 4.2% L-DOPA release was released from functionalized liposomes, compared to 68.4 ± 2.9% released from unfunctionalized liposomes. The presence of transferrin on functionalized liposomes might be the reason for the decrease in drug release as it reduces the liposomal membrane permeability, leading to a slowdown of releases. The in-vitro permeability of functionalized DA-loaded liposomes demonstrated a 5-fold increase in permeability compared to unfunctionalized liposomes [176]. The findings of this study highlight the positive potentials of surface modification for overcoming the challenge of the BBB, especially for polar molecules such as DA. 

## 5. Conclusions and Future Perspective

Although patients usually prefer long-acting DA agonists that can be taken once daily, in some patients, even these formulations require more than one dose per day to achieve therapeutic concentration. In addition to this, all the existing therapies available on the market are oral formulations, making them difficult to administer in patients with dysphagia. Therefore, sustained drug release such as long-acting injections may help in attaining constant plasma concentration, eliminate the need for multiple administration, and reduce side effects. The concept of an extended-release agonist is also strongly aligned with continuous dopaminergic stimulation theory. The idea that an extended-release formulation would lead to fewer plasma fluctuations and fewer motor difficulties is supported by this concept [91]. Numerous specialists have questioned this hypothesis; however, it has not been completely proven. 

Additionally, novel treatments developed for intranasal administration or subcutaneous infusion are also very inconvenient for patients. Intranasal formulations give immediate effect due to their ability to cross the BBB, but the effect is not prolonged due to the limitation in the dose and the effect of mucous clearance. Transdermal patches are also a good alternative, but they require administration in multiple doses of the drug, preventing these patches from being a convenient solution long term. Besides this, even though many liposomal systems provide high biocompatibility and good efficacy due to lipid constituents, a body of evidence has demonstrated their limited drug loading and short-term release for entrapped hosts. In addition to this, there are scale-up issues as this requires sterile conditions and process controls. In addition to this, during the development of new drug products, assuring the long-term stability of a liposomal formulation is critical. Furthermore, much research involving microspheres and nanoparticle-based DDSs conducted in-vivo evaluations on animals, and they need more clinical trials to prove their safety and efficacy, especially when the fate of nano-/microparticles in the body following administration remains a hot debate [177]. In addition to this, no guidelines have been published for the in-vitro release testing of sustained release parenteral formulations, making it difficult to test the release as it varies with the method used for the testing. More research needs to be conducted for achieving in-vitro release, which can correlate with release in vivo.

In addition to the traditional use of therapeutic molecules to treat PD, the use of stem cells to replace those cells destroyed by this disease might offer a breakthrough therapy in the management of PD. For example, in a pilot study conducted by Venkataramana and colleagues, seven PD patients received intracerebral transplantation with mesenchymal stem cells (MSCs) [178]. The MSCs were priorly isolated from the bone marrow of the patients and were cultured in a laboratory setting until the cells reached 90% confluency. All participants received a promising improvement of 38% on the Unified Parkinson’s Disease Rating Scale (UPDRS). The positive outcome of this pilot study has opened more doors to the use of stem cell therapy in addition to conventional drugs, moving toward an actual cure for PD. 

In the case of polymeric DDSs, the physiochemical properties of polymers should be carefully considered as they play an important role in overcoming multiple barriers in the brain, especially crossing the BBB. For example, soft polymeric particles have a longer circulation time and a lower elimination rate compared to hard particles. Therefore, particles with lower stiffness might have higher accumulation in the brain as the blood flow elevates [179]. In addition to this, polymers with higher molecular weights are more sufficient to reach cellular targets, have a longer circulation time, and tend to accumulate in the brain better [180,181]. However, it should be considered that despite having remarkable physicochemical characteristics in vitro, several nanosystems only have a modest in-vivo circulation time of less than a few hours. Therefore, to establish their physiological fate and accumulation properties, in-vivo tissue distribution and the accumulation of nanosystems should be evaluated [181]. 

With the advent of smart polymers, various DDSs have incorporated these materials to attain drug release triggered by various stimuli, such as temperature, light, and pH. Despite the outstanding control and release-on-demand of these systems, these DDSs are not without drawbacks. For example, burst release is a common issue with thermosensitive polymeric systems, caused by the slow sol-gel transition process in the body. To address this issue, several attempts have been made to optimize the ratio between hydrophilic and hydrophobic segments in the polymer chain [181]. For example, when linked to a peptide, the new triblock polymer system, namely PCL-PEG-PCL, has demonstrated a considerable decrease in initial burst release and a stable sustained-release pattern for more than a month [181]. Another challenge arrives from the degradability of overwhelmingly used materials. For example, highly charged and crosslinked smart polymers such as poly(acrylic acid) (PAA) are neither biodegradable nor recycled, posing serious environmental challenges [182]. To provide sustainable alternatives for such polymers, various naturally derived polymers can be made biodegradable by physical co-blending or via chemical modifications such as hydroxylation, alkylation, and graft copolymerization [183]. Another limitation of polymeric materials is the toxicity caused by the monomer aggregation or degradation process [184]. To address this issue, plasticizers can be added to a biopolymer to control its operative degradation, reducing the melt viscosity, temperature, pressure, and shear rate required to melt-form the polymers [185]. This can be achieved by including plasticizers in the backbone of polymers or adding them externally as discrete compounds [185]. In addition to this, the specific properties of polymers such as D,L-PLGA, specifically their composition and the chirality of the lactide in D,L-PLGA, are key factors affecting the degradation of drug carriers, and consequently the release of medicinal substances. 

As PD progresses rapidly, this disease has continued to impact the self-esteem, physical health, and independence of its sufferers, causing ramifications in their relationships with family and friends. To help alleviate their fear of the unknown, research groups need to take a proactive step toward advanced tools for better diagnosing and treating PD, and ultimately, toward developing prevention strategies against PD. Despite the early promising signs of nano-enabled formulations in the management of PD, bringing them to the market can be very difficult. Indeed, establishing these research programs for translational nanomedicines can be time-consuming, requiring resources and joint efforts from multi-national collaborators. Despite these challenges, with many funding opportunities and ongoing clinical trials in this field, there is a reason to hope that a cure for PD is coming soon. 

## Figures and Tables

**Table 1:** Currently available marketed products for PD.

Brand Name	Composition	Dosage Form	Drug-Release Mechanism	Limitation	Development Stage	Ref.
Sinemet CR^®^ Merck Sharpe and Dohme corp	L-DOPA, CD, hydroxypropyl cellulose, polyvinyl acetate-crotonic copolymer, magnesium stearate, and red ferric oxide	Controlled-release (CR) tablet	The drug is released through a combination mechanism of erosion and surface dissolution	Bioavailability is 30% less than IR tablet, which increases the dosage requirement	Approved/ Marketed	[32]
Parcopa^®^ Schwarz Pharma	L-DOPA, CD, aspartame, citric acid, cross povidone, magnesium stearate, mannitol, microcrystalline cellulose, natural and artificial mint flavor and sodium bicarbonate	Orally disintegrating	Tablet rapidly disintegrates on the tongue, releasing L-DOPA and CD	No substantial variations were observed in its motor advantages when compared to LD/CD IR	Approved/ Marketed	[33]
Stalevo^®^ Novartis Pharmaceutical	L-DOPA, CD, entacapone, corn starch, croscarmellose sodium, glycerol 85%, hypromellose, magnesium stearate, mannitol, polysorbate 80, povidone, sucrose, red iron oxide, and titanium dioxide	Immediate-release (IR), CR	The tablet is designed to release the three drugs at different rates and locations in the GIT, CD is immediately released followed by extended the release of L-DOPA in the small intestine, and finally delayed release of entacapone portion in the small intestine	Requires administration four times a day. Evokes a few motor complications	Approved/ Marketed	[34]
Rytary^®^ Impax pharmaceuticals	L-DOPA, CD, microcrystalline cellulose, mannitol, tartaric acid, ethyl cellulose, hypromellose, sodium starch glycolate, sodium lauryl sulfate, povidone, talc, methacrylic copolymers, triethyl citrate, croscarmellose sodium, and magnesium stearate	IR layer with delayed-release beads	The outermost layer disintegrates immediately providing the initial dose by dissolution, whereas the remaining layer releases the drug by diffusion	PD-induced gastroparesis might impact the effect of rotary movement	Approved/ Marketed	[35]
Duopa^®^ Abbvie Inc	L-DOPA, CD, carmellose sodium	Intestinal gel	The drug is released by diffusion and dissolution from the gel	Recommended for only 16 h. Adverse events related to the device are more likely to occur within the initial two weeks and are in line with the recognized complications of the PEG-J procedure [36]	Approved/ Marketed	[37]
Requip^®^ GlaxoSmithKline	Ropinirole, hypromellose, hydrogenated castor oil, carmellose sodium, povidone K29–32, maltodextrin, magnesium stearate, lactose monohydrate, anhydrous colloidal silica, mannitol, ferric oxide yellow (E172) and glycerol dibehenate	Prolonged-release tablet	The drug is released by diffusion and erosion where hypromellose acts as rate-controlling matrix	NA	Approved/ Marketed	[38]
Permax^®^ Eli Lilly and Amarin	Pergolide mesylate, croscarmellose sodium, iron oxide, lactose, magnesium stearate, and povidone	IR tablet	Once administered, the tablet disintegrates rapidly due to croscarmellose sodium after encountering gastric fluid	Adverse effects on heart valve	Withdrawn from market	[39]
Mirapex ER^®^ Boehringer Ingelheim, Pfizer	Pramipexole, hypromellose, corn starch, carbomer homopolymer, colloidal silicon dioxide, and magnesium stearate	ER tablet	Extended release was achieved by combining three polymers such as hypromellose, corn starch, and carbomer homopolymer, after encountering digestive fluid. Drug first dissolves from the surface followed by matrix swelling, slow diffusion of the drug, along with the erosion of the tablet	NA	Approved/ Marketed	[40]
Dostinex^®^ Pfizer	Cabergoline, leucine, and lactose	IR tablet	Tablet disintegrates rapidly once administered, releasing the drug, which is absorbed quickly into the blood circulation	NA	Approved/ Marketed	[41]
Eldepryl^®^ Somerset	Selegiline, citric acid, lactose, magnesium stearate, and microcrystalline cellulose	IR capsule	NA	NA	Approved/ Marketed	[42]
Zelapar^®^ Valeant pharmaceutical	Selegiline, gelatin, mannitol, glycine, aspartame, citric acid, yellow iron oxide, and grapefruit flavor	Orally disintegrating tablet	When the tablet is kept on the tongue, it disintegrates into small particles, which then dissolve in the saliva and are absorbed through the mucous membranes of the mouth	Can irritate the buccal mucosa	Approved/ Marketed	[43]
Apokyn Mylan Bertek	Apomorphine hydrochloride, sodium metabisulphite, sodium hydroxide, benzyl alcohol, hydrochloric acid	Subcutaneous injection	The drug is immediately absorbed into the bloodstream after administration of subcutaneous solution formulation (peak time 10–60 min)	Skin nodules at the site of injection	Approved/ Marketed	[34]
APO-go^®^ Pen Britannia Pharmaceuticals	Apomorphine, sodium bisulfite, hydrochloric acid	Subcutaneous injection	The drug is immediately absorbed into the bloodstream after administration of subcutaneous solution formulation	Skin nodules at the site of injection	Approved/ Marketed	[34]
Kynmobi Sunovion Pharmaceuticals Inc	Apomorphine, disodium EDTA, dihydrate, FDandC Blue #1, glycerol, glyceryl monostearate, hydroxyethyl cellulose, hypromellose, maltodextrin, (-)-menthol, pyridoxine hydrochloride, sodium hydroxide, sodium metabisulfite, sucralose, and white ink	Sublingual film	When kept under the tongue, the film disintegrates in 3 min, releasing the drug, which is rapidly absorbed through a thin mucous membrane under the tongue. Maximum concentration reached within 0.5 to 1 h	The problem of stomatitis at higher dose	Approved/ Marketed	[34]
Neupro^®^ UCB Pharma Limited	Backing film—aluminized polyester Drug matrix—rotigotine, ascorbyl palmitate, povidone, silicone adhesive, sodium metabisulfite, and dl-α-tocopherol Protective liner—transparent fluoropolymer-coated polyester film	Transcutaneous patch	45% of rotigotine is released within 24 h from the patch by diffusion	Requires replacement every 24 h	Approved/ Marketed	[34]
Treatments in Clinical Trials						
AP09004 Accordin pill™ Intec Pharma Ltd.	L-DOPA, CD	Gastro- retentive capsule	NA	Further studies are required to study its effectiveness in delaying the onset of motor complications	Completed Phase 2	[44]
DM-1992 Depomed Inc Acuform^™^ technology	L-DOPA, CD	Gastro- retentive bilayered tablet	NA	Not statistically significant	Completed Phase 2	[45]
XP21279 Xenoport Inc	Prodrug of L-DOPA, CD	Sustained-release bilayered tablet	NA	No difference in the reduction of off period	Completed Phase 2	[46]
V1512 Vernalis/Chiesi Farmaceutici SpA	L-DOPA methyl ester, CD	Effervescent tablet	NA	No significant difference in pharmacokinetics compared to that of a conventional tablet	Completed Phase 2	[47]
TRIGEL LobSor Pharmaceuticals AB	L-DOPA, CD and Entacapone	Intestinal gel	NA	A patient needs to be hospitalized for 3 days (expected), and it cannot be given at home	Completed Phase 1	[48]
CVT-301 Acorda Therapeutics Inc	L-DOPA	Dry powder inhaler	NA	Cough induction due to the inhalation of powder	Completed Phase 3	[49]
LY03003 Luye Pharma	Rotigotine	Sustained-release microsphere	NA	NA	Completed Phase 1	[50]
IPX203 Impax pharma	L-DOPA, CD	IR and ER capsule	NA	NA	Completed Phase 3	[51]
ABBV-951 AbbVie	L-DOPA phosphate, CD phosphate	Subcutaneous infusion system	NA	NA	Completed Phase 3	[52]
ND0612 Neuroderm Ltd.	L-DOPA, CD	Subcutaneous infusion	NA	Small transient papules at infusion sites	Phase 3 ongoing	[53]
Infudopa SubC Vastra Gotaland Region	L-DOPA	IV, SC	NA	NA	Completed Phase 1	[54]
ProNeura™ Titan Pharmaceuticals	Ropinirole	Subcutaneous implant	NA	NA	Terminated Phase 2	[55]
SPM 952 UCB Pharma Limited	Rotigotine	Intranasal	NA	NA	Completed Phase 2	[56]
Prestwick Pharmaceuticals, Schering AG	Lisuride	Transcutaneous patch	NA	Increased incidence of psychotic events	Completed Phase 2	[34]

NA = not available.

**Table 4:** Microsphere formulations developed since 2015 for the treatment of PD.

Drug/API	Method	Polymer	Advantages	Route of Administration	References
Pramipexole	W/O/W emulsion solvent evaporation	D,L- PLGA _50:50_ (M.w 10,000 Da)	Plasma concentration revealed drug release up to 2 weeks	Intramuscular	[158]
Rasagiline	W/O/W emulsion solvent evaporation	Polycaprolactone	In-vitro prolonged release up to 45 days was observed, whereas improved pharmacodynamics up to 30 days was observed after single-dose administration	Subcutaneous	[159]
GDNF	W/O/W emulsion solvent evaporation	Poly(ε-caprolactone) (M.w 45,000 Da)	In in-vitro release studies, GDNF was released from the microsphere for up to 25 days	-	[160]
L-DOPA, CD	Electrohydrodynamic co-jetting	D,L-PLGA _50:50_ (Intrinsic viscosity-0.67 g/mol), PLA	More than 90% of the drugs were released within 24 h	Oral	[161]
Ropinirole	O/O emulsion solvent evaporation	Eudragit RS	50% of the drug was released in 12 h following zero-order kinetics	Oral	[162]
GDNF	W/O/W emulsion solvent evaporative	D,L-PLGA _50:50_ (M.w 34,000 Da)	Single administration provided sustained motor improvement and dopaminergic function restoration	Stereotaxic implantation	[163]

## Data Availability

No new data was created in this manuscript.
